# Effects of ultrasound‑guided pericapsular nerve group block in combination with laryngeal mask airway on anesthesia, postoperative analgesia, and recovery of elderly patients with femoral neck fracture undergoing closed reduction and internal fixation

**DOI:** 10.20452/wiitm.2024.17886

**Published:** 2024-07-23

**Authors:** Rongen Qiu, Yunping Lan, Gang Liu, Ruifeng Zeng

**Affiliations:** Department of Anesthesiology, Quzhou Affiliated Hospital of Wenzhou Medical University, Quzhou People’s Hospital, Quzhou, China; Department of Anesthesiology and Perioperative Medicine, Second Affiliated Hospital of Wenzhou Medical University, Wenzhou, China

**Keywords:** anesthesia, fracture fixation, laryngeal masks, nerve block, ultrasound

## Abstract

**INTRODUCTION:**

Femoral neck fracture (FNF) frequently occurs in the elderly. FNF is usually treated surgically, but patients often have comorbidities, which make the surgery complicated and increase the intraoperative risk.

**AIM:**

We aimed to assess the effects of ultrasound‑guided pericapsular nerve group block (PENGB) coupled with laryngeal mask airway (LMA) general anesthesia on anesthetic efficacy in elderly patients suffering from FNF undergoing closed reduction and internal fixation (CRIF), as well as their postopera‑ tive analgesia and rapid recovery.

**MATERIALS AND METHODS:**

Control group (n = 47) and study group (n = 57) were established for FNF patients hospitalized between October 2022 and August 2023, based on different anesthesia modes. Ultrasound‑guided fascia iliaca compartment block (FICB) and LMA general anesthesia were offered to the control group, while the study group underwent ultrasound‑guided PENGB and LMA general anesthesia.

**RESULTS:**

In comparison with the study group, heart rate (HR) and mean arterial pressure rose significantly in the control group at the time of skin incision, 20 minutes intraoperatively, and at the end of the surgery, and HR also increased when the patients entered a postanesthetic care unit (*P* <0.05). The study group, in comparison with the control group, exhibited reduced serum neuropeptide Y and substance P levels at 12 and 24 hours postoperatively, and lowered Visual Analogue Scale scores at various time points following the operation (*P* <0.05). In comparison with the control group, the study group had shorter postoperative eye‑opening, extubation, and ambulation times (*P* <0.05)

**CONCLUSIONS:**

Ultrasound‑guided PENGB with LMA general anesthesia are effective in elderly patients with FNF undergoing CRIF, and can stabilize the intraoperative hemodynamic state.

## INTRODUCTION

Femoral neck fracture (FNF), especially caused by osteoporosis,^1^ frequently occurs in the elderly. FNF is usually treated surgically, but patients often have comorbidities, which make the surgery complicated and increase their intraoperative risk.^2^ Closed reduction and internal fixation (CRIF) is a common surgical option for FNF, but elderly patients may suffer from significant pain and delayed ambulation postoperatively, which increases the possibility of postoperative complications.^3^^;^^4^ Therefore, an anesthesia method with enhanced analgesic effect and rapid recovery is of considerable importance in CRIF.

Due to poor overall physical condition, elderly patients have low tolerance to general anesthesia and considerable intraoperative fluctuations in heart rate (HR) and mean arterial blood pressure (MAP), which result in a high surgical risk.^5^ Ultrasound guidance has advantages over traditional neurostimulators, such as high puncture success rate, low dosage, and accelerated recovery.^6^^;^^7^ Ultrasound‑guided fascia iliaca compartment block (FICB) can significantly relieve post–hip replacement pain by positioning and blocking the fascia iliaca compartment.^8^ Nevertheless, it is less effective in blocking partial lumbar plexus nerves innervating the hip joint, and may also affect postoperative muscle strength.^9^ In addition, pericapsular nerve group block (PENGB) can block some sensory nerves originating from the anterior and posterior areas of hip joints, improving the postoperative comfort of the patients.^10^ Meanwhile, laryngeal mask airway (LMA) anesthesia not only effectively maintains airway patency, but also helps prevent tracheal injury. In the elderly patients, LMA anesthesia enhances safety and postoperative recovery. ^11^

A combination of different anesthetic drugs and modes can significantly enhance the anesthetic effect.^12^ FNF is generally an intracapsular fracture, while partial lumbar plexus nerves, such as the obturator foramen, accessory obturator foramen, and femoral nerve are the main origin of sensory nerves innervating the anterior capsule of the hip joint. Using them as the joint analgesic targets may provide a better anesthetic effect.^13^ Until now, most works regarding PENGB are retrospective studies and case reports, which is why multicenter randomized controlled studies are necessary to clarify its analgesic effect and postoperative recovery.

## AIM

The study compared the anesthetic effects of ultrasound‑guided PENGB plus LMA general anesthesia and ultrasound‑guided FICB plus LMA general anesthesia on the postoperative analgesia and rapid recovery of elderly patients with FNF undergoing CRIF, aiming to provide valuable clinical evidence for future treatment.

## MATERIALS AND METHODS

### Materials

This study was approved by the ethics committee of the Quzhou People’s Hospital (QPH202210003), and written informed consent was obtained from all patients. A total of 104 FNF patients admitted to our hospital from October 2022 to August 2023 were recruited. The inclusion criteria were as follows: 1) FNF diagnosed by clinical imaging,

2) no contraindications for anesthesia, 3) successful CRIF in our hospital, 4) age between 60 and 80 years, 5) full consciousness and verbal contact postoperatively, and 6) access to complete clinical data.

The following exclusion criteria were employed: 1) a class III disease as per the American Society of Anesthesiologists criteria, 2) lack of tolerance to anesthetics, 3) puncture site infection, 4) significant abnormalities of the hematological system or the cardiocerebrovascular system or serious diseases of the heart, lung, liver or kidney, or 5) other lesions obviously affecting movement of the lower limbs. The patients were divided into a control group (ultrasound‑guided FICB plus LMA general anesthesia; n = 47) and a study group (ultrasound‑guided PENGB plus LMA general anesthesia; n = 57) according to different anesthesia modes.

### Anesthesia methods

The patients underwent 8 hours of fasting and 2 hours of water deprivation before anesthesia. They were placed in a supine position, and the puncture site was determined, followed by thorough disinfection and draping around the site. Lumify 2–5 MHz ultrasound probe (C5‑2, Philips, Amsterdam, the Netherlands) was covered with a sterile disposable sheath.

**Figure 1:**
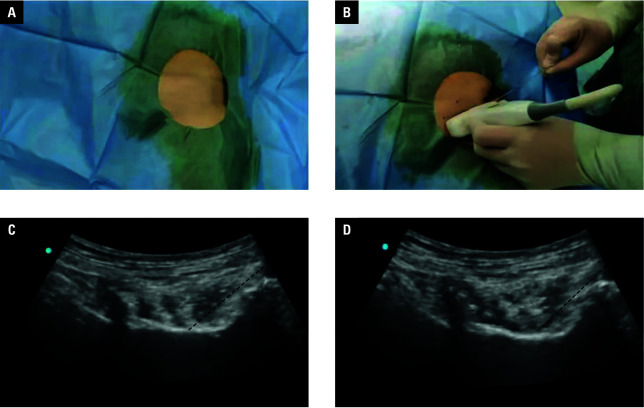
Procedure of ultrasound‑guided pericapsular nerve group block;

Ultrasound‑guided PENGB was performed in the study group (Figure 1). The ultrasound probe was placed at the inguinal ligament connecting the pubic tubercle with the anterior superior iliac spine, toward the lower eminence of the anterior margin of the ilium, clearly exposing the anatomical structure of the lower eminence and the iliopubic eminence. Then, the needle was inserted from outside to inside in the same plane as the ultrasound section until reaching the expected site (between the psoas major tendon and the pubic branch), followed by injection of 1 ml of normal saline to confirm the diffusion status of the drug liquid. Upon confirming the proper distribution range of the drug liquid in the target area, 30 ml of 0.25% ropivacaine (Jichuan Pharmaceutical Group Co., Ltd., Taixing, China) was injected.

**A** – left hip joint in the supine position; **B** – direction of the ultrasound probe; **C** – location of the needle tip (dashed line) between the psoas tendon and the pubic bone using the in‑plane method; **D** – slight lifting of the psoas tendon marking adequate fluid diffusion (dashed line)

Ultrasound‑guided FICB was performed in the control group. The ultrasound probe was placed about one‑third of its length away from the center of the lateral inguinal ligament. After the fascia iliaca covering the fusion surface of the iliacus and psoas major muscle was observed, the needle was inserted in the same way as in the study group until reaching the expected site (fascia iliaca compartment), and normal saline was also injected. Upon confirming the presence of normal saline in the potential fat space, 30 ml of 0.25% ropivacaine were injected.

The procedure of LMA general anesthesia is described below. Following the nerve block, the patients in both groups were given LMA general anesthesia with an injection containing 0.3 mg/kg etomidate (Jiangsu Nhwa Pharmaceutical Co., Ltd., Xuzhou, China), 0.15 mg/kg cisatracurium besylate (Sichuan Baili Pharmaceutical Co., Ltd., Chengdu, China), 10 mg of dexamethasone sodium phosphate (Yunnan Longchuan Zhangfeng Pharmaceutical Factory, Longchuan, China), and 0.25–0.5 g/kg sufentanil citrate (Langfang Branch of Sinopharm Group Co., Ltd., Langfang, China), at an oxygen flow rate and tidal volume of 2 l/min B – direction of the ultrasound probe; C – location of the needle tip (dashed line) between the psoas tendon and the pubic bone using the in‑plane method; D – slight lifting of the psoas tendon marking adequate fluid diffusion (dashed line) and 6‑8 ml/kg, respectively. With the respiratory rate set at 9–12 times/min, the expiratory time was twice the inspiratory time. Propofol injectable emulsion (Xi’an Libang Pharmaceutical Co., Ltd., Xi’an, China) was pumped continuously intraoperatively at 4–6 mg/kg/h to maintain anesthesia.

### Evaluation of hemodynamic variations

Both groups were assessed for MAP and HR prior to anesthesia induction, at the time of skin incision, 20 minutes intraoperatively, at the end of the operation, and at the time of entering the postanesthetic care unit (PACU).

### Detection of pain mediator levels

The levels of serum neuropeptide Y (NPY) and substance P (SP) were measured before the anesthesia, and 12 and 24 hours postoperatively. Blood samples were collected at the specified time points, and centrifuged at 3000 rpm for 10 minutes. Then the NPY and SP supernatant levels were measured with SN‑682B gamma radioimmunoassay counter (Shanghai Institute of Nuclear Research, Chinese Academy of Sciences, Shanghai, China) and radioimmunoassay kits (Linco Research, St. Charles, Missouri, United States), respectively.

### Pain assessment

Pain degree was assessed at 2, 4, 8, and 12 hours postoperatively in both groups using the modified Visual Analogue Scale (VAS).^14^ The patients marked their degree of pain on a straight, horizontal line at 0–10 cm (0–10 points). The higher the score, the stronger the pain.

### Time and quality of postoperative recovery

The postoperative eye‑opening, extubation, and ambulation times were subjected to intergroup comparisons. Functional status of all patients was assessed by using the Quality of Recovery‑15 scale (QoR‑15) at 1 day preoperatively and 1, 2, and 3 days postoperatively.^15^ The QoR‑15 consists of 15 items in 5 dimensions, with a total score of 150 points (0–10 points for each item). The lower the score, the worse recovery quality.

### Statistical analysis

Data were examined using SPSS 22.0 software (IBM Inc., Armonk, New York, United States), and expressed as numbers and percentage. They were compared using the χ^2^ test or the χ^2^ test with continuity correction. Measurement data were described by mean and SD and subjected to intergroup and intragroup comparisons with the independent‑sample *t* test and paired‑sample *t* test, respectively. Repeated measures analysis of variance was performed for intergroup comparison at multiple time points. The differences were treated as significant at a *P* value below 0.05.

## RESULTS

### Patient characteristics

A total of 104 patients participated in the study according to the screening criteria, with 57 and 47 participants allocated to the study group and the control group, respectively. The general characteristics in both groups were comparable (*P* >0.05) (TABLE 1 ).

**TABLE 1:** General characteristics of the patients

Parameter		Study group (n = 57)	Control group (n = 47)	χ^2^/Z/t^a^	*P *value
Sex, n (%)	Men	35 (61.4)	26 (55.32)	0.393	0.53
	Women	22 (38.6)	21 (44.68)		
Age, y		67.82 (8.34)	67.83 (8.14)	0.006	0.99
Body mass index, kg/m^2^		21.87 (2.68)	21.63 (2.66)	0.439	0.66
American Society of	I	24 (42.11)	21 (44.68)	0.04	0.84
Anesthesiologists class, n (%)	II	17 (29.82)	13 (27.66)		
	III	16 (28.07)	13 (27.66)		
Operation time, min		148.17 (18.41)	143.51 (19.05)	1.265	0.2

Data are provided as mean and SD unless stated otherwise.

**a** Count data were compared using the χ^2^ test or the χ^2^ test with continuity correction. Measurement data were subjected to intergroup comparison with the independent‑sample *t* test. Repeated measures analysis of variance was performed for multigroup comparison.

### Hemodynamic indicators at various time points

Differences in HR and MAP were clear between the groups and at diverse time points (*P* <0.001). The parameters first increased and then declined in both groups, with some differences being significant (*P* <0.05). The intergroup differences in HR and MAP before the induction and in MAP at the time of entering the PACU were not significant (*P* >0.05). Contrary to the study group, HR and MAP in the control group dropped considerably at the time of skin incision, 20 minutes intraoperatively, and at the end of the operation (*P* <0.05), and HR rose at the time of entering the PACU (*P* <0.05) (TABLE 2 ).

**TABLE 2:** Hemodynamic indicators at various time points

Parameter	Heart rate, bpm					Arterial pressure, mm Hg				
	Before induction	At the time of skin incision	20 min intraoperatively	At the end of the operation	At the time of entering PACU	Before induction	At the time of skin incision	20 min intraoperatively	At the end of the operation	At the time of entering PACU
Study group (n = 57)	77.35 (7.31)	79.08 (9.89)^e^	80.25 (6.73)^ae^	79.73 (7.74)^e^	80.15 (8.22)^e^	115.06 (11.35)	115.29 (9.79)^e^	117.4 (8.1)^e^	14.4 (7.58)^ce^	114.44 (10.44)
Control group (n = 47)	76.95 (7.17)	87.39 (11.56)^a^	91.29 (6.62)^ab^	90.14 (6.56)^a^	85.15 (8.22)^acd^	115.76 (12.03)	129.76 (12.95)^a^	131.48 (12.97)^a^	127.27 (11.88)^a^	118.72 (12.75)^bcd^
*F*_intergroup_/ *P*_intergroup_^f^	91.88 / <0.001					91.75 / <0.001				
*F*_time_/*P*_time_^f^	17.71 / <0.001					12.69 / <0.001				
*F*_interaction_/ *P*_interaction_^f^	8.594 / <0.001					8.602 / <0.001				

Data are provided as mean and SD.

**a** *P* <0.05 vs before induction in the same group

**b** *P* <0.05 vs at the time of skin incision in the same group

**c** *P* <0.05 vs 20 min intraoperatively in the same group

**d** *P* <0.05 vs at the end of the operation in the same group

**e** *P* <0.05 vs control group at the same time point

**f** Measurement data were subjected to intergroup and intragroup comparisons with the independent‑sample *t* test and paired‑sample *t* test, respectively. Repeated measures analysis of variance was performed for comparison at different time points.

Abbreviations: PACU, postanesthetic care unit

### Pain mediators at various time points

Differences in NPY and SP levels were perceptible in the 2 groups and at different time points (*P* <0.001). The levels of the mediators increased postoperatively in both groups (*P* <0.05). No major differences were observed in NPY and SP content between the groups prior to anesthesia (*P* >0.05), but the study group had lower NPY and SP content than the control group at 12 and 24 hours postoperatively (*P* <0.05) (TABLE 3).

**TABLE 3:** Levels of pain mediators at various time points

Parameter	Serum neuropeptide Y, ng/l			Substance P, ng/l		
	Before anesthesia	12 h postoperatively	24 h postoperatively	Before anesthesia	12 h postoperatively	24 h postoperatively
Study group (n = 57)	95.77 (11.66)	105.06 (14.23)^ac^	120.25 (15.88)^ab^	40 (5.76)	52.69 (7.03)^ac^	62.68 (8.22)^abc^
Control group (n = 47)	96.77 (11.37)	124.55 (17.31)^a^	142.74 (19.03)^ab^	40.05 (6.27)	59.74 (8.52)^a^	73.51 (10.29)^ab^
*F*_intergroup_/ *P*_intergroup_^d^	69.87 / <0.001			45.84 / <0.001		
*F*_time_/*P*_time_^d^	140.9 / <0.001			339.7 / <0.001		
*F*_interaction_/ *P*i_nteraction_^d^	15.37 / <0.001			12.8 / <0.001		

Data are provided as mean and SD.

**a** *P* <0.05 vs before anesthesia in the same group

**b** *P* <0.05 vs 12 h postoperatively in the same group

**c** *P* <0.05 vs control group at the same time point

**d** Measurement data were subjected to intergroup and intragroup comparisons with the independent‑sample *t* test and paired‑sample *t* test, respectively. Repeated measures analysis of variance was performed for comparison at different time points.

### Pain degree at different time points

Intergroup comparison at various time points showed differences in the VAS score (*P* <0.001). The VAS score first increased and then decreased in both groups, with some differences being significant (*P* <0.05). In comparison with the control group at diverse time points after the operation, the study group presented lower VAS score (*P* <0.05) (TABLE 4).

**TABLE 4:** Postoperative recovery time

Parameter	Visual analogue scale score, points			
	2 h postoperatively	4 h postoperatively	8 h postoperatively	12 h postoperatively
Study group (n = 57)	1.83 (0.43)	2.02 (0.52)^ad^	3.39 (0.69)^ad^	1.85 (0.44)^cd^
Control group (n = 47)	2.25 (0.55)	3.18 (0.91)^a^	4.03 (1.13)^ab^	3.31 (0.93)^ac^
*F*_intergroup_/ *P*_intergroup_^e^	166.6 / <0.001			
*F*_time_/*P*_time_^e^	96.86 / <0.001			
*F*_interaction_/ *P*i_nteraction_^e^	11.12 / <0.001			

Data are provided as mean and SD.

**a** *P* <0.05 vs 2 h postoperatively in the same group

**b** *P* <0.05 vs 4 h postoperatively in the same group

**c** *P* <0.05 vs 8 h postoperatively in the same group

**d** *P* <0.05 vs control group at the same time point

**e** Measurement data were subjected to intergroup and intragroup comparisons with the independent‑sample *t* test and paired‑sample *t* test, respectively. Repeated measures analysis of variance was performed for comparison at different time points.

### Postoperative recovery time and quality

In the control group, postoperative eye‑opening, extubation, and ambulation times were shorter than in the study group (*P* <0.05) (TABLE 5). The groups differed in their QoR‑15 score at various time points (*P* <0.001). The score increased in both groups postoperatively (*P* <0.05). It showed no difference between the groups at 1 d preoperatively (*P* >0.05), but was slightly higher in the study group, as compared with the control group at 1, 2, and 3 days postoperatively (*P* <0.05) (TABLE 6).

**TABLE 5:** Quality of Recovery‑15 score at different time points

Parameter	Postoperative eye‑opening time, min	Postoperative extubation time, min	Postoperative ambulation time, d
Study group (n = 57)	6.83 (1.13)	11.07 (2.53)	4.18 (1.14)
Control group (n = 47)	7.44 (1.24)	12.67 (4.19)	5.14 (1.65)
*t *test^a^	2.622	2.402	3.497
*P *value	0.01	0.02	0.001

Data are provided as mean and SD.

**a** Measurement data were subjected to intergroup comparison with the independent‑sample *t* test.

**TABLE 6:** Quality of Recovery‑15 score at different time points

Parameter	QoR‑15 score, points			
	1 d preoperatively	1 d postoperatively	2 d postoperatively	3 d postoperatively
Study group (n = 57)	104.43 (7.72)	121.71 (4.76)^ad^	127.47 (3.31)^abd^	138.47 (3.85)^abcd^
Control group (n = 47)	105.33 (6.96)	117.37 (5.08)^a^	125.78 (4.63)^ab^	131.48 (3.57)^abc^
*F*_intergroup_/ *P*_intergroup_^e^	34.97 / <0.001			
*F*_time_/*P*_time_^e^	619.4 / <0.001			
*F*_interaction_/ *P*i_nteraction_^e^	10.99 / <0.001			

Data are provided as mean and SD.

**a** *P* <0.05 vs 1 d preoperatively in the same group

**b** *P* <0.05 vs 1 d postoperatively in the same group

**c** *P* <0.05 vs 2 d postoperatively in the same group

**d** *P* <0.05 vs control group at the same time point e Measurement data were subjected to intergroup and intragroup comparisons with the independent‑sample *t* test and paired‑sample *t* test, respectively. Repeated measures analysis of variance was performed for comparison at different time points.

Abbreviations: QoR‑15, Quality of Recovery‑15 score

## DISCUSSION

In this study, the control group patients had higher HR and MAP than the study group individuals at the time of skin incision, 20 minutes intraoperatively, and at the end of the operation. Higher HR was also measured in the control group than in the study group at the time of entering the PACU, suggesting that ultrasound‑guided PENGB plus LMA general anesthesia can better stabilize the hemodynamic status during CRIF for elderly patients with FNF. Probably, PENGB can more directly affect the nerve structure related to the hip joint during CRIF, and block the nerves more directly related to the hip joint, such as the obturator foramen, the accessory obturator foramen, and the femoral nerve, thereby achieving more effective local anesthesia. In contrast, FICB mainly blocks the iliac nerve and its branches.^16^ Therefore, PENGB can offer more efficient pain control intraoperatively, thereby maintaining patient comfort, reducing stress reactions, and stabilizing the hemodynamic status.^17^

Additionally, the study group patients had significantly lower NPY and SP contents than the control group individuals at 12 and 24 hours postoperatively, and remarkably decreased VAS scores at different time points postoperatively, suggesting that ultrasound‑guided PENGB plus LMA general anesthesia exerted an obvious analgesic effect in the elderly patients with FNF following CRIF. Pain of the fracture end, surrounding soft tissues, and even of the whole body is one of the most significant postoperative discomforts in fracture patients, and it often occurs 6 hours postoperatively, involving an increase in the levels of pain transmitters, mainly NPY and SP neuropeptides.^18^ NPY plays a key role in pain control in fracture patients by inducing vasoconstriction and excitability in pain areas, thus enhancing pain perception.^19^ Released by the central nervous system and peripheral nervous system, SP is implicated in the process of pain conduction.^20^ PENGB has a stronger inhibitory effect on the pain nerve directly related to the hip joint,^21^ and pain transmitters increase postoperatively due to inflammation.^22^ PENGB is superior to FICB in suppressing proinflammatory factors, such as tumor necrosis factor‑α and interleukin‑1β,^23^ so it may also indirectly control NPY and SP levels through its anti‑inflammatory effect.

In this study, local anesthetic drugs were injected around the nerve trunk and its main branches to produce an anesthetic effect in both the study and the control group. The block effect was affected by many factors, including nerve fibers of different thicknesses, dosage and concentration of the anesthetic drugs, and accuracy of determining the injection site.^24^ The reason for the lower VAS score in the study group can be attributed to the characteristics of PENG. Although Aδ fibers are contained in PENG, iliac nerve and its branches, PENG also contains thinner nerve C fibers.^25^ Generally speaking, the PENG nerve fibers are thinner, so it has a lower requirement for the effective concentration of anesthetic drugs. In contrast, the injection site in FICB is far from the target nerve, and the obturator nerve may cross and branch with other structures in FIC, so anesthetic drugs cannot fully act on the obturator nerve, resulting in an insufficient block. Moreover, at 1, 2, and 3 days following the operation, postoperative eye‑opening, extubation, and ambulation times were significantly reduced, and QoR‑15 scores distinctly increased in the study group vs the control group, suggesting that ultrasound‑guided PENGB plus LMA general anesthesia contributed to rapid recovery.

The reason of this is that PENGB can stabilize the hemodynamics intraoperatively, which can promote postoperative eye‑opening and extubation to some extent. Although PENGB can block the nerve supply of the hip joint by affecting joint branches, it does not affect the associated skin and muscle innervation of the hip joint, so the quadriceps muscle strength is retained during PENGB,^26^ and this helps shorten the ambulation time. In addition, the study group had mild postoperative pain, felt more comfortable, and gastrointestinal motility was promoted by earlier ambulation, so the nutritional status improved rapidly, thereby increasing the QoR‑15 score.

### Limitations

Limitations of the study include small sample size, and the fact that all samples were from the orthopedics department of our hospital.

## CONCLUSIONS

In conclusion, ultrasound‑guided PENGB plus LMA general anesthesia are effective in elderly patients with FNF undergoing CRIF. The method allows clinicians to stabilize the hemodynamic status, achieve a good postoperative analgesic effect, shorten the recovery time, and enhance quality of the recovery. To further clarify the anesthetic effect of ultrasound‑guided PENGB plus LMA general anesthesia on elderly patients with FNF during CRIF, larger‑sample multicenter studies are required.
